# Coming out of isolation: impacts of COVID-19 on physical activity, diet, mental well-being, and sleep over time

**DOI:** 10.3389/fpsyg.2024.1462297

**Published:** 2024-11-13

**Authors:** Joel Billings, Allison Kwesell, Stephanie Cosby, Shuyang Lin

**Affiliations:** ^1^Department of Emergency, Disaster, and Global Security Studies, Embry–Riddle Aeronautical University, Daytona Beach, FL, United States; ^2^Pratt Institute, New York, NY, United States

**Keywords:** COVID-19, panel longitudinal, mental well-being, sleep, physical activity, diet, quarantine

## Abstract

**Introduction:**

The COVID-19 pandemic lockdown significantly disrupted daily routines and impacted physical activity, diet, mental well-being, and sleep. This mixed-methods study investigates these changes over three periods—pre-pandemic, pandemic onset, and one-year post-onset—to understand their causes and inform public health policy for improved resilience during future crises.

**Methods:**

A mixed-methods study was conducted with 34 US participants who completed open-ended qualitative questions and quantitative assessments in May 2020 and May 2021. Participants were recruited via social media from 10 states with high COVID-19 cases. Quantitative data categorized health changes (positive, negative, or no change), while qualitative data were analyzed thematically to explore reasons for these changes and uncover interrelationships among health behaviors.

**Results:**

Quantitative results showed that, during the pandemic onset, most participants experienced negative changes in diet, mental well-being, and sleep, while physical activity varied. By one-year post-onset, improvements were noted across all health pillars, with mental well-being and sleep showing the most significant positive changes. Thematic analysis of qualitative data revealed stress, anxiety, and personal motivations as key drivers of health behaviors. Participants’ narratives provided a deeper understanding of these shifts that a Likert-style survey alone could not capture, highlighting the interconnectedness of health pillars.

**Discussion:**

The findings demonstrate the importance of addressing mental well-being to improve overall health resilience. Public health interventions should prioritize mental health and consider the interrelated nature of health behaviors. The mixed-methods approach combined quantitative trends with qualitative insights, offering a comprehensive understanding of why health changes occurred, providing actionable guidance for future public health policy.

## Introduction

The purpose of this research is to assess the impact of the lockdown from COVID-19 on physical activity, diet, mental well-being, and sleep across three periods: before the pandemic (winter 2019), at the onset (May 2020), and 1 year later (May 2021) in the US. As COVID-19 spread globally, varying responses emerged at national and state levels. In the United States, lockdowns were first mandated in California on March 19, 2020, with continued safety precautions like social distancing recommended by the CDC (2020). As a result, many lived in some form of isolation with interrupted social routines, which have been documented to impact mental health including stress, anxiety, depression, and post-traumatic stress injury, among others (Agha, 2021; Xiong et al., 2020).

The impact of COVID-19 has also been documented in other areas of health. Existing studies have documented that reduced physical activity, poor diet, and disrupted sleep during lockdowns negatively impacted mood in the United Kingdom and Scotland (Ingram et al., 2020; López-Bueno et al., 2020). While much disaster research focuses on structural damages, the social-psychological effects and long-term health outcomes may persist longer (Hasegawa et al., 2016; Bromet, 2011; Brooks et al., 2020). However, there is limited understanding of personal perspectives on how health behaviors evolved throughout the pandemic. This study aims to fill this gap by exploring individuals’ management of daily routines, physical activity, diet, mental well-being, and sleep. The findings can inform public health policies and enhance resilience in future crises, aiding emergency management and resource prioritization.

Physical activity, diet, mental well-being, and sleep are fundamental pillars of health (Duncan et al., 2019). In a study of military personnel, Szivak and Kraemer (2015) found that while inherently stressful experiences may result in comorbidities, those who prioritize pillars of health are more physiologically prepared, resilient, and better able to cope with adversity. This suggests that compromising any one of these pillars, or living with an imbalance among the four, may influence acute and chronic health consequences and impair resilience to trauma from disasters.

Furthermore, the effects of COVID-19 and interrupted social routines have been documented in studies of stress and anxiety (Menzies and Menzies, 2020; Rosen et al., 2020), drug and alcohol use (Fatke et al., 2020), sleep (Gupta et al., 2020), physical activity (López-Bueno et al., 2020), changes in employment (Weerakoon et al., 2021), and food and product disruption in supply chains (Hobbs, 2020). Mandatory confinement during COVID-19 has been shown to negatively impact physical activity, sleep quality, and well-being (Martínez-de-Quel et al., 2021). Whilst these trends have been observed, little is known about the underlying reasons for these negative effects, which is why this study focuses on individuals’ perceptions of the causes behind changes in their physical activity, diet, mental well-being, and sleep.

## Pillars of health

### Physical activity

The U.S. Department of Health and Human Services recommends that adults receive at least 150 min of moderate or 75 min of vigorous exercise weekly (HHS, 2018). However, mandatory lockdowns for non-essential workers encouraged many to limit activity outside the home and even prevented access to fitness facilities. While social isolation has been associated with reduced physical activity (Robins et al., 2018; Schrempft et al., 2019), COVID-19-related research is varied. Some found an increase in physical activity (Di Renzo et al., 2020), whereas others found decreased physical activity (López-Bueno et al., 2020; Yang and Koenigstorfer, 2020; Ingram et al., 2020). However, these studies often do not address why individuals increased or decreased their physical activity during this period, leaving an incomplete understanding of the factors that influenced behavior changes. This study aims to explore the reasons behind changes in physical activity during the pandemic and how these behaviors evolved over time.

### Nutrition

Poor nutrition influences obesity, diabetes, cardiovascular disease, and skeletal and joint disorders (Ross et al., 2014) and can exacerbate COVID-19 complications. For example, Butler and Barrientos (2020) suggest that obesity and type 2 diabetes may increase the risk of COVID-19 complications. In contrast, those who eat more vegetables and fruits have been found to have a reduced risk of contracting COVID-19, and those who contract the virus tend not to develop severe symptoms of COVID-19 (Merino et al., 2021). The pandemic had a significant impact on diet, as people’s eating habits were influenced by various factors, including lockdowns, stress, and changes in food accessibility. Researchers found that more processed foods were consumed globally, especially during the onset of the pandemic (Ruíz-Roso et al., 2020; Cummings et al., 2022). At the same time, people confined to their residences increasingly took up cooking at home (Sarda et al., 2022), which may have led to healthier diets for some. Despite these documented changes, little research has focused on understanding why these dietary behaviors shifted throughout the pandemic.

### Mental well-being

Pandemic-related studies have documented adverse mental health effects, including increased stress, anxiety, depression, and post-traumatic stress injury (Agha, 2021; Xiong et al., 2020). In a study that assessed emotional well-being associated with specific daily activities, Lades et al. (2020) found that physical activity, pursuing hobbies, and spending time with children increased positive effects, whereas activities like homeschooling and reviewing COVID-19 information were associated with negative effects. Furthermore, decreased mental well-being has been found to have a consequential impact on children and families, especially in families who experience lower income, racism, and marginalization (Prime et al., 2020). Those with preexisting mental health disorders typically experienced worsening symptoms during the pandemic (Hao et al., 2020). While isolation experienced during the pandemic affected mental well-being, less is known about the underlying causes for these changes and how they evolved as the pandemic progressed.

### Sleep

While physical activity, nutrition, and mental well-being have long been understood to be pillars of health, sleep was only recently recognized as a pillar of health by Duncan et al. (2019). Sleep has many bidirectional relationships with other health domains; sleep loss can cause acute effects, including decreased reaction speed and cognitive performance (Kryger et al., 2022). If left untreated, sleep loss can increase the risk of severe health consequences (Costa, 1996), including cardiovascular disease (Mullington et al., 2009), obesity, and diabetes (Knutson and Van Cauter, 2008; Yaggi et al., 2006), as well as depressed immune system function (Dinges et al., 1995; Luyster et al., 2012), which has been found to increase vulnerability to COVID-19 (Mello et al., 2020). While the National Sleep Foundation recommends adults receive 7–9 h of sleep each night, research has found that 40 percent of the general population and healthcare workers reported sleep disturbances during the pandemic, with those infected by COVID-19 experiencing a greater prevalence of sleep-related disturbances (Jahrami et al., 2021).

In each pillar, research has shown the impact of COVID-19. However, less is understood about why people experience changes in each pillar and why their behaviors might evolve throughout the pandemic. This leads to two research questions: (1) Why did individuals experience changes in health and daily responsibilities during the onset of the pandemic? (2) Why did individuals experience changes in health and daily responsibilities 1 year after the onset of the pandemic? By focusing on these four pillars, the study aims to shed light on how these interrelated domains were affected by the pandemic and to understand the individual-level causes behind these changes. This understanding is crucial for informing public health policy and enhancing resilience in future public health crises.

## Materials and methods

In May 2020, we conducted a pilot study using a long-form questionnaire to explore the stressors induced by the pandemic (Kwesell et al., 2022). The data indicated that participants were experiencing a range of external stressors, some related to direct fears about contracting the virus, while others stemmed from major lifestyle changes brought on by the pandemic. These stressors impacted important areas of health, such as physical activity, diet, mental well-being, and sleep. Moreover, participants’ ways of coping with the collective trauma of the pandemic further influenced these health behaviors. These preliminary findings highlighted the need for deeper investigation. As a result, we conducted a second questionnaire to retrospectively examine habits related to physical activity, diet, mental well-being, sleep, and daily responsibilities across three time periods: pre-pandemic, onset, and one-year post-onset. The study received IRB approval.

While health is a multi-dimensional concept that can be framed through various models, such as the biopsychosocial paradigm or the World Health Organization’s holistic definition of health, this study focuses on four key pillars: physical activity, nutrition, mental well-being, and sleep. These pillars are particularly relevant in understanding the behavioral health changes that occurred during the COVID-19 pandemic, as they encompass physical and mental health dimensions commonly affected by social isolation and lifestyle disruptions.

### Participant recruitment

To solicit participation, social media advertisements were posted on Facebook pages in New York, New Jersey, Michigan, Massachusetts, Pennsylvania, California, Louisiana, Illinois, Florida, and Texas (the 10 states reporting the most cases of COVID-19 at the time of posting according to the Johns Hopkins Coronavirus Resource Center). The advertisement requested people to join a research project about living in isolation (quarantine or lockdown) during the COVID-19 pandemic or share the advertisement with other individuals, thereby employing a snowball sampling method. The advertisement included an email address to contact the research team for those interested in participation. Those who expressed interest were sent a link to informed consent and the first questionnaire. In total, 57 participants from 19 states completed a long-form questionnaire in May 2020. Participants were contacted again 10 months after the first data collection to solicit interest for the second data collection. Thirty-seven agreed to participate, 34 of which completed the questionnaire. While the study did face some attrition, a 60% cooperation rate with repeat participants after 1 year is considered a feat, as past research has found greater attrition rates during vulnerable times (Rothenbühler and Voorpostel, 2016; Ginn et al., 2017).

### Data collection

Assessing phenomena over time through longitudinal research has been found to strengthen causal inferences (Ployhart and Vandenberg, 2010). Data collection occurred in May 2020, and the second round occurred in May 2021, employing a panel research design. In both rounds, participants received an email with a link to an online open-ended questionnaire that remained open for 1 week. Additionally, a reminder email was sent 3 days after the participant received the original email. Questionnaires inquired about stressors induced by the pandemic, the frequency with which they left their home, the reasons they went outside, the amount of time away from their home, where they lived, employment status, changes in employment, and their connectedness to others.

Unique to the second-round questionnaire included questions on physical activity, diet, sleeping habits, and mental well-being. For each area, participants were asked to explain their habits (1) before the pandemic (winter of 2019), (2) at the onset of the pandemic (May 2020), and (3) one year after the beginning of the pandemic (May 2021). Participants were also asked to explain the ways in which their overall lifestyle responsibilities and activities changed in the same sequence design as mentioned above, in addition to indicating their current vaccination status.

### Data analysis

A team of four researchers first read the narratives from each participant to explore and familiarize themselves with the data. This initial reading phase was crucial for understanding the data before beginning the coding process (Maietta, 2006). Based on the patterns observed in the participants’ responses, we categorized them into three groups: those who reported positive change, no change, or negative change for each pillar. This approach was informed by much of the existing COVID-19 research, which focused on reported increases or decreases in the pillars, and we aimed to understand the reasons behind any changes in health behaviors. These categories allowed us to systematically assess the reasons for the changes within each group. After the coding scheme was applied, the four researchers met for a series of meetings to resolve any discrepancies, ensuring inter-rater reliability. While 34 participants completed the online questionnaire, some did not answer all questions or respond to each time period (i.e., habits before the pandemic, during onset, and one-year post), so results are reported as percentages of responses received.

The mixed-methods approach in this study involved analyzing both qualitative and quantitative data. While the qualitative data provided in-depth insights into participants’ health behaviors and motivations, the quantitative data revealed broader trends across the sample. To ensure the reliability of the findings, a process of triangulation was employed, whereby the results from both data sets were compared and cross-validated. This allowed us to identify areas of convergence between the narrative explanations and the quantitative trends, further strengthening the study’s conclusions. For example, themes related to changes in mental well-being and physical activity were consistently supported by both qualitative reflections and quantitative data, confirming the interconnectedness of these health pillars during the pandemic.

## Results

Table 1 provides a detailed summary of the 34 participants’ characteristics. The cohort included 27 women and 7 men, with an average age of 47 years (range 21–80). Participants were recruited from 14 states, with the majority maintaining stable employment throughout the study. Notably, 85% of participants had received at least one vaccination dose by May 2021.

**Table 1:** Participant characteristics.

Participants	34 (27 women, 7 men)
Mean age (range)	47 (21–80)
Vaccination status (May 2021)	29 (85%) Vaccinated
Job status change (due to COVID-19)	8 (24%) lost their job
Home state in US (*n*)	AZ (1), CO (7), FL (2), GA (3), IL (2), NC (2), NJ (1), NY (1), OH (2), OR (5), SC (1), TN (4), TX (2), WA (1)
Occupation (*n*)	Education (6) Entertainment/Photography/Journalism (5) Real estate (4) Medical/human health services (3) Self-employed (4) Services/Sales (3) Retired/unemployed (2) Stay-at-home parent (2) Student (2) Technology (1) Faith-based (1) Law (1)
Frequency of leaving the house during lockdowns	< 7 times a week: 13 (43%) 7 times a week: 15 (50%) 8–14 times a week: 1 (3%) > 14 times a week: 1 (3%) Nonresponse: 4

### Overall longitudinal changes in health behaviors

#### Physical activity

At the onset of the pandemic, 38% of participants increased their physical activity, attributing it to having more time and the necessity to maintain sanity through exercise. However, 32% reported a decline due to restricted access to fitness facilities. By 1 year later, 52% had further increased their physical activity, often citing the adoption of new hobbies such as hiking or home workouts.

#### Diet

During the onset, 42% of participants reported negative changes in diet, often due to stress-eating or reduced motivation to cook healthy meals. Conversely, 18% noted improvements as they cooked more at home. By 1 year later, 39% improved their diet, driven by health awareness and better access to healthy foods.

#### Mental well-being

Initially, 62% of participants experienced a decline in mental well-being due to anxiety and isolation. Over time, many adopted coping strategies such as reducing media consumption, engaging in hobbies, and seeking therapy. 1 year later, 57% reported improvements, highlighting the role of reflection and new routines.

#### Sleep

At the pandemic’s onset, 45% reported worsening sleep due to anxiety and disrupted routines. Despite flexible schedules, few saw improvements early on. A year later, 35% experienced better sleep, often attributed to medication and established routines.

### Changes from pre-pandemic to onset (May 2020)

By May 2020, participants had spent an average of 45 days in isolation. Most left their homes for exercise, groceries, work, and managing stress by changing their environment (e.g., going for a drive). The pandemic brought changes in daily responsibilities, including increased home chores and childcare, with some participants sharing more responsibilities with partners due to concerns about virus exposure. For example, one participant noted, “We do not have a house cleaner anymore, and we do it ourselves because we do not want anyone in our home.” Participants also experienced new sources of stress as role shifts occurred in the absence of external support, such as school closures and work-from-home demands.

**Figure 1:**
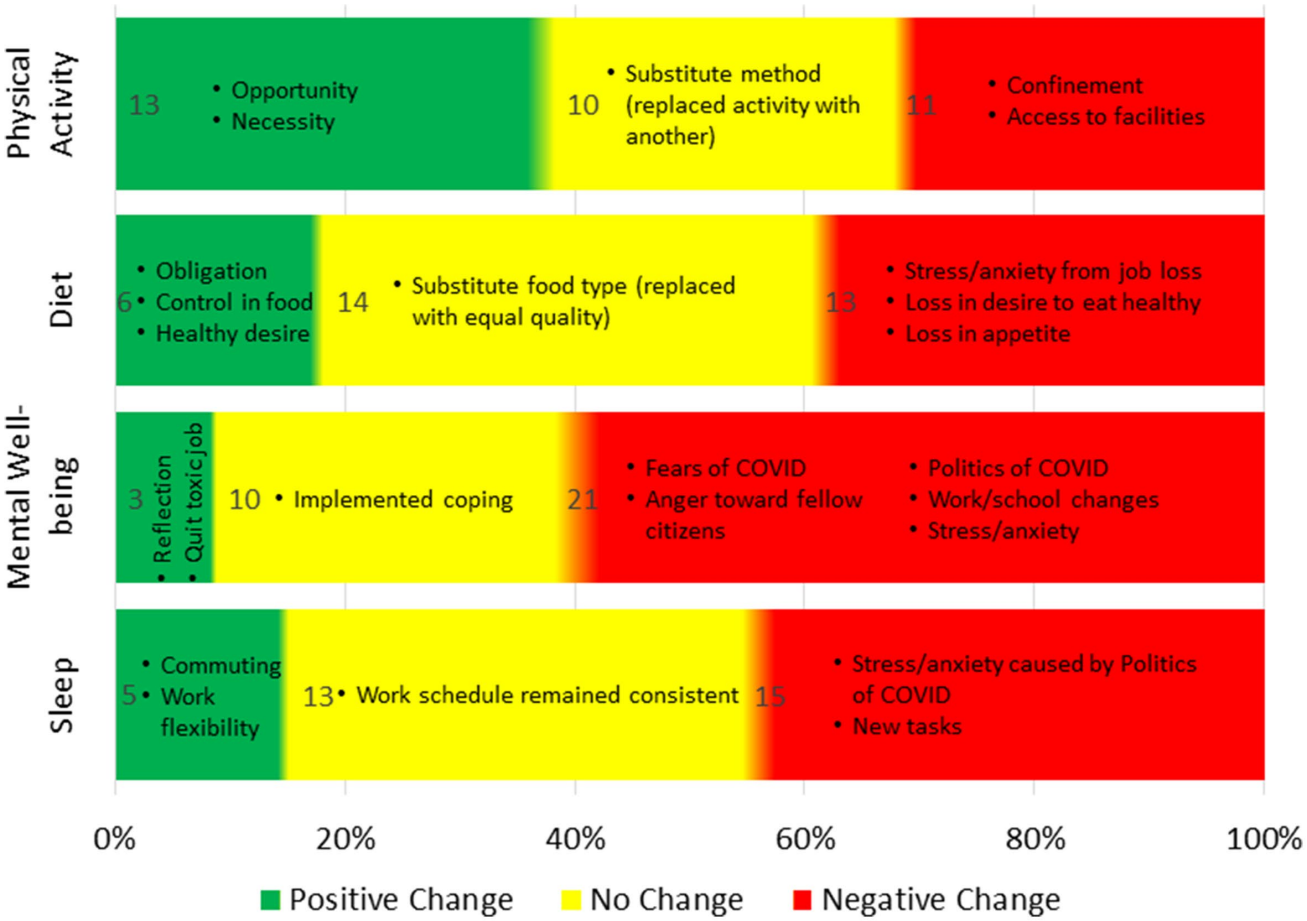
Changes in health before pandemic to onset.

The majority of participants experienced health changes (Figure 1), with most reporting negative impacts on diet, mental well-being, and sleep, while physical activity changes were more balanced. Mental well-being was most affected, with 62% reporting negative changes.

#### Physical activity

Physical activity changes were relatively evenly split: 38% reported a positive change, 29% no change, and 32% a negative change. Positive changes were often driven by new habits or mental health coping strategies, such as hiking or exercising to manage stress. One participant explained, “I HAD to exercise for sanity. [I] HAD to get outside. The only place I could go.” Others cited more time at home as an opportunity for increased activity. Some participants did not change their activity levels but modified their methods, like shifting from gym workouts to home-based exercises. Those who experienced negative changes often attributed it to confinement or loss of access to facilities, with one participant saying, “I had not really moved my body in weeks, and it had really affected me.”

#### Diet

Dietary changes were primarily negative, with 18% reporting positive changes and 39% experiencing negative shifts. Those with positive changes cited cooking at home and gaining control over their food choices as key factors. For example, one participant mentioned, “I began to cook again during the pandemic, and it was really good for my mental health.” Negative changes were often tied to stress and anxiety, leading to overeating or increased alcohol consumption. One participant described gaining 30 pounds due to anxiety-induced eating. Others reported a loss of appetite, with one noting, “I had no motivation [during] the early pandemic and no appetite.”

#### Mental well-being

Mental well-being was significantly impacted, with 62% reporting a decline. Anxiety, fear of death, political stress, and changes in work and school routines were major contributors to negative mental health. One participant reflected on the amplification of pre-existing concerns, saying, “The pandemic was kind of an amplifier for concerns/doubts I already had about finances, marriage, and life in general.” A few participants, however, found positive mental health changes through self-reflection or leaving toxic environments, with one stating, “Getting out of that environment was the healthiest thing to ever happen to me.” Lastly, one participant found that online socializing helped them remain consistent in their relationship: “For most of the time, online socializing helped my relationship with my guy.”

#### Sleep

Sleep was also affected, with 45% reporting negative changes and only 15% experiencing improvements. Those with positive changes credited work-from-home flexibility for allowing more sleep, while those with negative changes pointed to stress, anxiety, and political unrest as major disruptors. Some participants noted using medications or sleep aids, while others highlighted late-night worries or rumination about the pandemic and politics, with one participant sharing, “Politics—particularly the election and aftermath—caused me more sleep loss than COVID.”

### Changes from onset (May 2020) to one-year-post pandemic (May 2021)

One year after the pandemic’s onset, several participants experienced shifts in their lives. Nearly 26% of participants had moved, and 17% of those moves were due to job losses. Eighty-six percent had received at least one dose of the COVID-19 vaccine, and 44% reported feeling more connected to others compared to 23% during the onset. Daily responsibilities also evolved as participants adapted to changing routines at home, returning to in-person work or school, or starting new businesses. For some, this led to a balance in their home and work life, while others noted that they had “adjusted to a new rhythm.”

**Figure 2:**
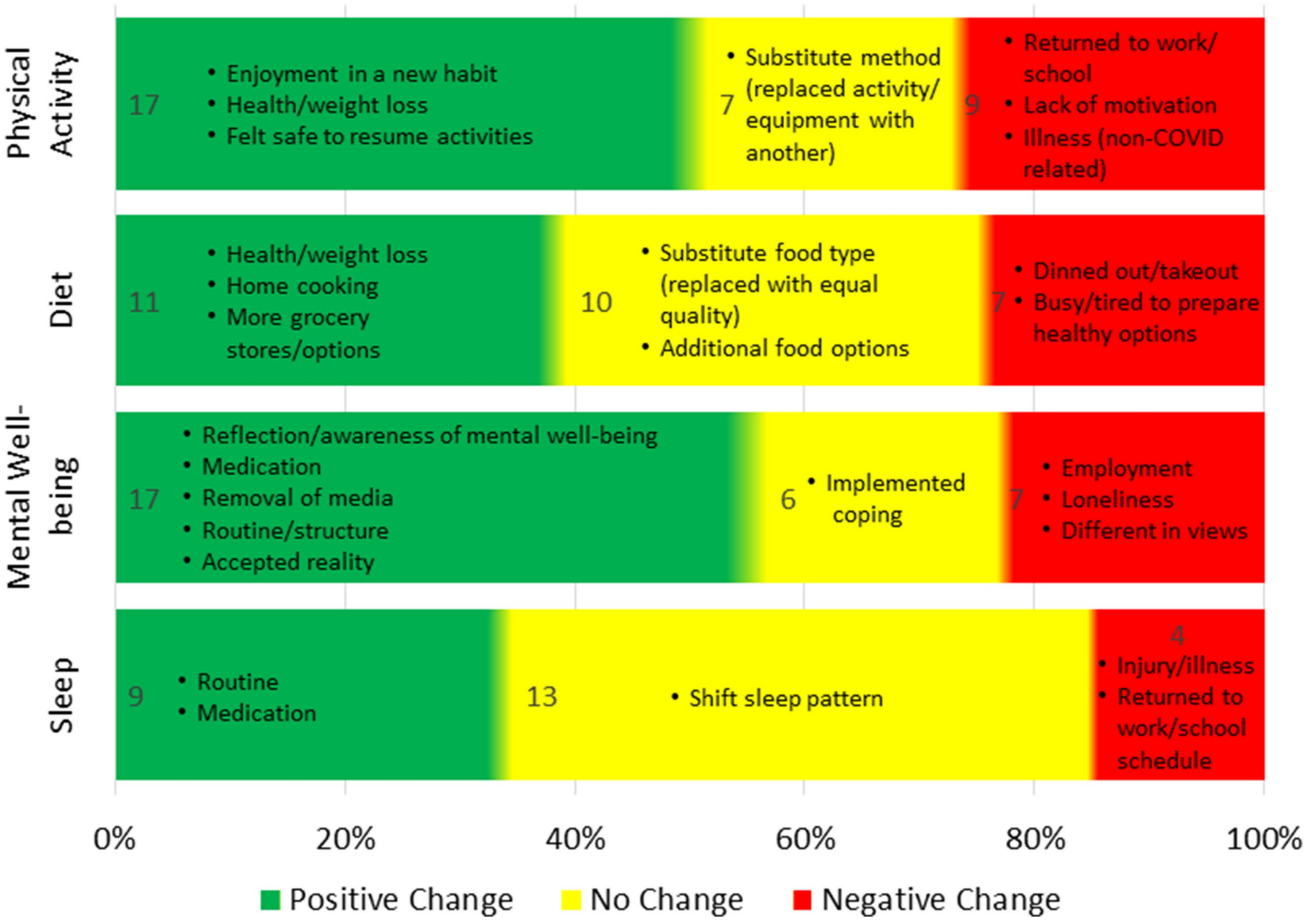
Changes in health between onset of pandemic to 1-year post.

In terms of health, most participants experienced changes in physical activity (79%), diet (64%), mental well-being (80%), and sleep (50%). Positive changes in mental well-being were the most notable (Figure 2).

#### Physical activity

One year after the pandemic’s onset, 52% of participants reported positive changes in physical activity, motivated by new hobbies, weight loss, or feeling safe enough to resume previous activities. For example, one participant stated, “I feel better when I exercise more,” while others mentioned becoming more aware of their sedentary habits and taking steps to improve their health. Twenty-one percent of participants reported no change in physical activity, with some adapting their methods but not increasing activity levels. Those who experienced negative changes (27%) cited returning to work or school, lack of motivation, or non-COVID-related factors such as injury, illness, or pregnancy. One participant explained, “During our time at home, we walked every day, but now, with new jobs and moving, we are not walking as much.”

#### Diet

One year after the pandemic’s onset, 39% of participants reported positive changes in diet, driven by motivations such as weight loss, home cooking, and new grocery store options. Some participants became more health-conscious, stating they were focused on “losing the pandemic pounds,” while others enjoyed cooking at home, with one noting, “I actually like the food I cook better than restaurant food now.” Thirty-six percent of participants reported no change in their diet, though some switched food choices without altering quality. Among the 25% who reported a negative change, reasons included fatigue from maintaining healthy habits or turning to takeout due to being too busy or tired. One participant explained, “Feeding four people every day is exhausting,” while others noted that dining out felt like a treat after long periods of isolation.

#### Mental well-being

Since the onset of the pandemic, 57% of participants reported a positive change in mental well-being, driven by factors such as self-reflection, developing routines, seeking therapy or medication, and reducing media consumption. Several participants mentioned that the pandemic offered an opportunity for introspection, helping them to find peace and improve their outlook on life. One participant explained, “The pandemic gave me a pause, to stop the rat race and heal.” Others noted that reaching rock bottom pushed them to seek professional help, with one stating, “I started taking medication for my ADHD, and this is the happiest I’ve ever been.” For 20% of participants, there was no change in mental well-being, with some noting that their mental health had remained stable, whether positive or negative, throughout the pandemic. Meanwhile, 23% reported a decline in mental health, citing feelings of loneliness, frustration with differing views, and employment struggles. One participant expressed, “The loneliness has grated on me, and I’ve gotten in my head about certain friendships,” while others noted heightened anger and difficulty adjusting to life post-pandemic.

#### Sleep

One year after the pandemic’s onset, 35% of participants reported positive changes in their sleep habits, often attributed to creating new routines or seeking medical advice. One participant explained, “I made an effort to work on my sleep habits, setting a morning and night routine, and it paid off.” Others found that consulting a doctor improved their sleep quality. Meanwhile, 50% of participants reported no change in their sleep, with most saying their sleep patterns remained generally consistent, whether good or poor. Some noted slight shifts in their routines, such as going to bed earlier, but no significant changes. For the 15% who reported a negative change, causes included returning to normal work or school schedules, injury, illness, or other personal health issues. One participant noted, “As things open up more, I get way less sleep and less quality sleep,” while another cited struggles with cancer as impacting their rest. However, it is not clear whether the latter was related to cancer treatment, concerns about health, or other factors associated with the pandemic or the easing of restrictions.

## Discussion

The COVID-19 pandemic caused widespread lockdowns, disrupting social routines and creating uncertainty. This study explored changes in health behaviors across three time periods. Initially, the pandemic negatively impacted diet, mental well-being, and sleep, consistent with findings from Ingram et al. (2020) and López-Bueno et al. (2020). Some participants adopted coping strategies such as reduced media consumption and increased exercise; findings that are similar to Bhattacharjee and Acharya, (2020), which helped maintain stability in certain health pillars. Notably, physical activity was the only pillar where more participants reported positive changes during the onset.

One year after the onset of the pandemic, there was a reported overall improvement in physical activity, diet, mental well-being, and sleep compared to the pandemic onset. Many participants adjusted to new routines, indicating resilience and adaptability. The results suggest that changes in behaviors were interconnected, with mental well-being and sleep being critical contributors to overall health. Poor mental health negatively affected sleep, which in turn influenced physical activity and diet. This aligns with the work of Duncan et al. (2019), who emphasize the bidirectional relationship between sleep and mental health, as well as the impacts of sleep disturbances on physical activity and diet. Their work underscores the importance of sleep as a foundational pillar of health, with disruptions in one area having far-reaching effects on others. Our findings support this model by demonstrating how improvements in sleep led to better mental well-being, which in turn influenced healthier behaviors in physical activity and diet.

Beyond the research questions, the study reveals a process of reflection and adaptation. Participants reflected on their situations, modified their routines, and subsequently changed their behaviors, as illustrated by Prime et al. (2020). This adaptive process underscores the importance of mental well-being in managing health behaviors during crises. The findings suggest that public health policies should prioritize mental health support to enhance overall resilience and well-being in future emergencies.

### Processing COVID-19

The onset of the pandemic caused significant social disruption, leading many participants to reflect on their lives as a coping mechanism. Among the four health pillars, mental health was most affected, with participants frequently citing fears of the virus, mental exhaustion, and loneliness, aligning with findings from Agha (2021) and Xiong et al. (2020). Data from a year later suggests that participants began processing COVID-19 through activities that brought emotional relief, such as journaling, meditation, or taking time for introspection. One participant described how this period allowed them to focus on what truly mattered, saying, “I had time to think about the things that are important, which I had neglected before.” Another participant remarked, “the pandemic gave me a pause, to stop the rat race.” Others described feelings of “languishing”—a state of being stuck or stagnant during the pandemic, not a coping strategy itself but a recognized emotional response to prolonged stress. The process of reflection and the development of coping strategies, such as therapy or routine-building, were instrumental in moving participants from languishing toward self-improvement, as suggested by Bhattacharjee and Acharya (2020). This evolution from feeling stuck to making meaningful changes illustrates how participants used reflection as a means of adaptation.

Reflection emerged as a recurring theme across all health pillars. For instance, one participant found that increased alone time led to better self-understanding, while another became more intentional about their relationships. Some participants even realized their work environments were toxic, prompting resignations, a trend seen during the “great resignation” (Demirkaya et al., 2022). Through reflection, many participants began to develop new routines as they processed the ongoing pandemic and its disruptions.

### Managing new routines

As the pandemic progressed, individuals had to adapt to changing circumstances by developing new routines. Many participants reported that lockdowns forced them to manage tasks differently, often resulting in positive adjustments. For example, the closure of fitness facilities pushed some to develop better workout plans or engage in outdoor activities, a trend also noted in previous research (López-Bueno et al., 2020). Similarly, home cooking became a necessity for many, which led to healthier eating habits. This mirrors findings by Ammar et al. (2020), who reported that increased time at home allowed individuals to take control of their diet and develop healthier routines.

Parents, in particular, faced added responsibilities, including overseeing their children’s education and meals, which exacerbated stress levels, especially among those working from home. Prime et al. (2020) also highlighted this dual burden, noting that parents struggled to balance work, caregiving, and household tasks, particularly during periods of school closures. However, while some parents managed to adapt to these new roles, others found it difficult, reflecting the variability in how families coped with pandemic-related changes.

The effects of working from home were mixed. While some participants enjoyed the flexibility and reduced commute time, others found it challenging due to distractions and a lack of structure. This aligns with the work of Xiao et al. (2021), who noted that working from home during the pandemic provided both benefits and challenges, depending on individuals’ ability to manage time effectively. As restrictions eased and businesses and schools reopened, many participants reported a shift in routines once again. For example, cooking at home became less frequent as dining out resumed, though participants often noted improvements in physical activity as they felt safer engaging in outdoor activities—an observation consistent with studies that noted a resurgence in outdoor activities as restrictions were lifted (Di Renzo et al., 2020).

Interestingly, despite increased flexibility in schedules at the pandemic’s onset, few participants reported improvements in sleep. Anxiety, fear, and rumination continued to disrupt sleep for many, consistent with the findings of Jahrami et al. (2021), who observed widespread sleep disturbances during the early phases of the pandemic. However, as time passed, many participants experienced improvements in their sleep patterns, often attributed to the development of new routines and, in some cases, medical interventions. This suggests that while initial responses to the pandemic may have been fraught with stress and anxiety, participants were able to regain some stability in their health behaviors as the situation evolved.

### Changes experienced and interrelationships of pillars

Participants reported a wide range of behavioral changes during the pandemic, which were influenced by individual circumstances such as family responsibilities, personal motivations, and access to resources. For example, while one parent increased physical activity out of necessity due to childcare responsibilities, another saw the pandemic as an opportunity to focus on health improvement. This variation in responses aligns with research by Ammar et al. (2020), who demonstrated that individuals adapted differently to confinement, with some embracing new habits and others facing barriers due to environmental constraints. These findings underscore the personalized nature of health behavior changes during crises.

Stress and anxiety emerged as major factors negatively affecting participants’ mental well-being and sleep, supporting earlier research by Ingram et al. (2020), and López-Bueno et al. (2020), who found that anxiety heightened during the pandemic and contributed to sleep disturbances. In addition to the general increase in stress and anxiety reported by participants, individuals with pre-existing vulnerabilities may have experienced even higher levels of anxiety during the pandemic. This is consistent with findings by Heinze et al. (2022), who observed heightened state anxiety and poor sleep quality in individuals with disabilities and visual impairments, underscoring the compounded impact of the pandemic on those with additional health challenges. Moreover, mental health challenges were also exacerbated by the constant influx of distressing COVID-19 news, as well as political tensions, illustrating the complex external pressures that participants faced. Not only did these stressors affect mental well-being, but they also had cascading effects on other health behaviors, particularly diet. Participants reported stress-eating, reduced appetite, or irregular eating patterns, consistent with findings by Ruíz-Roso et al. (2020), who observed increased consumption of ultra-processed foods among confined populations.

On the other hand, improvements in physical activity and diet were frequently associated with positive impacts on mental well-being and sleep, highlighting the interconnected nature of these health pillars. For instance, one participant explained that cooking at home during the pandemic provided a sense of control and improved their mental health, echoing the findings of Di Renzo et al. (2020), who reported that home-based routines helped mitigate some of the negative psychological effects of confinement. Another participant noted that increased physical activity improved their sleep, reinforcing the bidirectional relationship between exercise and sleep, as described by Duncan et al. (2019). This interrelationship between physical and mental health is particularly important in times of crisis, as improvements in one domain can lead to enhancements in others, creating a positive feedback loop that contributes to overall well-being.

The holistic nature of health is further emphasized by the fact that participants who experienced improvements in one health pillar often saw corresponding gains in others. For example, participants who reported better physical activity habits often noted concurrent improvements in their diet and mental well-being, suggesting that interventions targeting one aspect of health can have broader effects. Conversely, declines in mental well-being or sleep tended to negatively impact participants’ ability to maintain healthy physical activity or diet, as also highlighted by Jahrami et al. (2021), who found that sleep disturbances during the pandemic were closely tied to stress and overall health decline. This reinforces the need for a multi-dimensional approach to public health, where the interdependencies between different health behaviors are acknowledged and addressed collectively.

### Public health implications

These findings are particularly relevant for shaping public health strategies in the wake of COVID-19. Building resilience across all health pillars, with a special focus on mental well-being and sleep, is crucial for fostering long-term health stability in the face of future crises. Mental well-being, in particular, serves as a foundation for sustaining healthy behaviors in other areas, and its neglect can lead to widespread deterioration in physical activity, diet, and sleep patterns. Public health initiatives should therefore prioritize integrated interventions that support mental health, while simultaneously promoting regular physical activity, healthy eating, and proper sleep hygiene to help individuals maintain balanced, resilient lifestyles during times of uncertainty.

### Limitations

This study contains a few noteworthy limitations. While recruiting using social media allowed for the collection of a geographically diverse sample across 14 states, the online questionnaire design limited the ability to probe for additional details regarding changes in health behaviors. This limitation likely contributed to some missing data, where participants indicated a shift in behavior but did not fully explain their reasons for the change. However, the open-ended nature of the questions still allowed for rich qualitative data collection.

Additionally, the self-selecting nature of the sample may have attracted participants who were more likely to have experienced issues or changes in their health behaviors during the pandemic, potentially leading to bias in the data. While this is a common challenge in qualitative research, the diversity of the sample helped provide a range of perspectives. Another consideration is the retrospective nature of the participants’ reflections on their behaviors before the pandemic, which may have introduced recall bias. However, participants were asked to reflect on significant lifestyle changes, which are typically easier to recall accurately.

Moreover, the relatively small sample size (34 participants) may limit the generalizability of the findings, as it primarily provides in-depth insights rather than broad trends. While this is typical of qualitative research, caution should be exercised when interpreting the results for larger populations. Furthermore, the absence of a control group restricts our ability to compare behavior changes with a non-pandemic population, though the aim of this study was to explore lived experiences rather than establish direct cause-effect relationships.

It is also important to note that this research was part of an ongoing study, meaning participants were drawn from a pre-existing cohort rather than recruited based on a new research question. This approach allowed for continuity in data collection and deeper insight into behavior changes over time, though it may have led to some bias in the sample as participants already had established connections to the study. Finally, although we sought to capture individual perceptions of health changes, the survey design did not quantify the extent of these changes, which could have provided further insights for public health providers in understanding the impact on each health pillar.

In the United States, public health strategies such as social distancing and lockdowns were implemented at varying levels across states, but overall, these measures were generally less strict and shorter in duration compared to other countries like Australia and the United Kingdom, which enforced more stringent, longer-lasting restrictions. The health impacts observed in this study may reflect the relatively moderate interventions seen in the US context. A comparison with countries under stricter measures could provide deeper insights into how the intensity and duration of public health restrictions shape health behaviors and outcomes.

## Conclusion

As the impact of COVID-19 on human health continues to be studied, this mixed-methods study explored directional changes in physical activity, diet, mental well-being, and sleep behaviors, alongside participants’ explanations for these changes. The qualitative insights gathered through open-ended responses offered a deeper understanding of the underlying motivations and emotional factors influencing these behavioral shifts, such as the importance of reflection, adaptation, and coping strategies in navigating the pandemic’s challenges.

Key findings highlight how stress and anxiety negatively impacted multiple health pillars, while positive changes in one domain (e.g., physical activity or diet) often had beneficial ripple effects on others, such as mental well-being and sleep. These insights underscore the need for public health policies to go beyond isolated health interventions and embrace holistic approaches that simultaneously address mental, physical, and emotional health.

Future research should focus on longitudinal studies that continue to track these behavioral changes and explore how they evolve in the post-pandemic period. Additionally, understanding the specific mechanisms that facilitate resilience in times of crisis can inform more targeted interventions aimed at improving both individual and community-level health outcomes during future global emergencies.

## Data Availability

The raw data supporting the conclusions of this article will be made available by the authors, without undue reservation.
